# Vitamin D as a Risk Factor for Multiple Sclerosis: Immunoregulatory or Neuroprotective?

**DOI:** 10.3389/fneur.2022.796933

**Published:** 2022-05-16

**Authors:** Sara E. Gombash, Priscilla W. Lee, Elizabeth Sawdai, Amy E. Lovett-Racke

**Affiliations:** ^1^Department of Neuroscience, The Ohio State University, Columbus, OH, United States; ^2^Department of Microbial Infection and Immunity, The Ohio State University, Columbus, OH, United States

**Keywords:** multiple sclerosis, vitamin D, neuroprotection, immune regulation, oxidative stress

## Abstract

Vitamin D insufficiency during childhood has been linked to the development of multiple sclerosis (MS), typically an adult-onset inflammatory demyelinating disease of the central nervous system (CNS). Since vitamin D was known to have immunoregulatory properties on both innate and adaptive immunity, it was hypothesized that low vitamin D resulted in aberrant immune responses and the development of MS. However, vitamin D receptors are present on many cell types, including neurons, oligodendrocytes, astrocytes and microglia, and vitamin D has profound effects on development and function of the CNS. This leads to the possibility that low vitamin D may alter the CNS in a manner that makes it vulnerable to inflammation and the development of MS. This review analysis the role of vitamin D in the immune and nervous system, and how vitamin D insufficiency in children may contribute to the development of MS.

## Introduction

The importance of vitamin D (VitD) in health was formally recognized in 1922 when it was determined that cod liver oil and sunlight cured rickets (1, 2). Our understanding of the role of VitD has expanded well beyond bone health with the observation that VitD receptors (VDR) are widely expressed on many cell types in many tissues. VitD is a fat-soluble vitamin with limited availability in foods. Thus, the predominant source of VitD is synthesis in the skin after sun exposure. VitD is a steroid hormone that regulates numerous genes important in cell differentiation, proliferation and homeostasis. Unfortunately, VitD deficiency is prevalent worldwide, with infants, pregnant women, the elderly, and dark-skinned individuals being the most affected (3). There is substantial literature describing the importance of VitD as an immune regulator with a growing body of evidence on the importance of VitD on the development and function of the nervous system. While VitD deficiency has been correlated with a variety of human diseases, there is substantial evidence that VitD deficiency, particularly in children, may be a major risk factor for the development of multiple sclerosis (MS), which is typically diagnosed in young adults. Historically, it has been postulated that low VitD may be promoting a dysregulated and/or hyperactivated immune system that leads to CNS inflammation. However, the fact that low VitD in children appears to be more closely associated with MS than other autoimmune diseases suggests that VitD insufficiency may be playing an important role in the central nervous system (CNS), making it more vulnerable to inflammation. Thus, VitD deficiency in children may be contributing to the risk of developing MS as an adult by dysregulation of genes in both the immune system and CNS.

## Multiple Sclerosis

Multiple sclerosis (MS) is an inflammatory, demyelinating disease affecting an estimated 2.8 million people worldwide (4). Clinically, MS is characterized by relapsing and progressive neurological dysfunction. In most patients, the disease begins with episodes of neurological dysfunction followed by complete or partial remission— the relapsing/remitting form of the disease (RRMS). In some RRMS patients, the disease is later transformed into uninterrupted progression of neurological deficits — the secondary progressive phase of the disease (SPMS). Other patients' disease initiates with a slow, progressive accumulation of neurological dysfunction — primary progressive multiple sclerosis (PPMS) (5). Pathologically, MS is characterized by focal plaques of demyelination with activated microglia and abundant peripheral inflammatory cells in the CNS. The cause of the disease is unknown and therapies are limited to disease modifying medications that reduce the number inflammatory lesions and slow disease progression.

### Geographical Distribution of MS

Many decades ago it was found that MS has the lowest frequency along the equator, and increases prevalence with increasing latitude (6). The relationship between latitude and MS risks has been observed in several studies. MS frequency among French farmers displayed a north-south gradient and was inversely correlated with exposure to sunlight, though the gene pools and life styles of individuals were broadly comparable (7). UK migrants who live in Tasmania in the south had greater MS frequency than those that migrated to tropical Queensland (8). Although MS risks seem to decrease with migration from high to low latitudes (9), the timing of migration has critical effects on this change. Migration studies have shown that people who are younger than 15 years at the time of migration tend to adopt MS risks of the country to which they migrate, whereas those who are older than 15 years retain similar incidence as their country of origin. A recent study in New Zealand confirmed the latitude gradient, but also found that the prevalence gradient was strongest at birth (10). A comprehensive meta-analysis of 94 studies published through 2018 confirmed the latitude gradient in MS (11). Analysis of sun exposure based on age found that living in an area with high UV-B before MS onset was associated with a 45% lower risk of MS, and a 51% reduction in risk when living in a medium to high UV-B area from 5-15 year of age (12). Overall, the risk of developing MS is largely determined before the age of 15 years (13–17) or at least within the first two decades (18), suggesting a role for the environment in modifying MS risks during childhood and adolescence.

### Genetic and Environmental Risk Factors in MS

The cause of MS is unknown. It remains unclear what triggers the immune system to attack the myelin sheath. Twin studies reveal that genetic factors have important roles in MS risk. The rate of concordance for MS among monozygotic twins is 25–40%, which is much higher than the 5% concordance rate among dizygotic twins (19). Genome-wide sequencing studies have further identified human leukocyte antigen (HLA) class II exerting the strongest association with MS risks (20, 21). On the other hand, the concordance rate among monozygotic twins is not 100%, which means MS risks are not fully determined by genetics.

Unquestionably, the environment is also influential on disease susceptibility. Although the identity of environmental factors involved in MS is not yet unequivocally known, accumulating evidence lends strong supports to several candidates: Epstein-Barr virus (EBV) infection, cigarette smoking and VitD. The relationship between EBV seropositivity and MS risks is now firmly established (*p* < 10–23). Virtually all (99.5%) patients with MS are seropositive for antibodies directed against EBV (22). A recent study analyzed multiple environmental factors that may contribute to MS risk, including VitD levels and EBV antibody titers (23). EBV antibody titers were significantly higher in MS patients and there was an inverse relationship between VitD levels and EDSS, yet no correlation between VitD levels and EBV antibody titers. The prevalence of EBV infection is high (94%) in age/gender-matched controls, so the vast majority of infected individuals do not develop MS, which suggests that EBV infection may be a necessary contributing factor to MS risk but not a cause of MS. For cigarette smoking, a positive association between smoking before age of onset and MS risks is found in some case-control studies (24, 25). Individuals with RRMS have an increased risk of developing SPMS if they have ever smoked, compared with non-smokers (26). These factors— genetics, EBV infection and smoking— may work interactively to determine MS susceptibility, but none of them can fully explain the geographic variations in MS frequency and the changes in risk that occur with migration.

### Mouse Model of MS

Much of our fundamental understanding of MS is based on observations in rodent models of MS such as experimental autoimmune encephalomyelitis (EAE). EAE resembles MS in both clinical and pathological aspects (27, 28). Susceptibility to EAE and clinical course vary among strains of mice. For instance, B10.PL and SJL/J mouse strains are two of the more commonly used susceptible strains, whereas BALB/c is much less susceptible (29). SJL/J mice develop a relapsing-remitting disease that can transition into a progressive disease over time, closely resembling RRMS and the transition to the SPMS form (28). C57B/6 mice have become the most utilized EAE model due to the availability of genetically modified mice on the C57B/6 background that allows for defining the role of specific molecules in CNS autoimmunity. The downside of using the C57B/6 mouse model is that disease course and inflammatory components of the lesion have distinct differences from the human condition. Instead, C57B/6 mice develop a rapid-onset, chronic neurological disease without relapses. Furthermore, antibodies and neutrophils contribute significantly to lesion formation which is not typical of MS (30, 31). Some have speculated that C57B/6 EAE may actually be a better model for neuromyelitis optica (NMO), a rare autoimmune neurodegenerative disease very similar in phenotype to MS in which antibodies to aquaporin 4 and neutrophils are known to contribute to the formation of demyelinating lesions (32). These different EAE models have contributed to our understanding of CNS autoimmunity, yet we should be cognizant of how they may or may not reflect MS.

EAE can be induced by several methods. Active induction of EAE is done by direct immunization with myelin proteins or peptides emulsified in complete Freund's adjuvant. The myelin protein and/or peptide used differs because of variations in MHC between strains of mice. Passive induction of EAE is done by transfer of activated myelin-specific CD4 T cells into a naïve mouse. The myelin-specific CD4 T cells can be generated by immunization with a myelin protein/peptide, followed by removal of the draining lymph nodes, reactivation of the myelin-specific T cells *in vitro*, and injection of the myelin-specific T cells into naïve mice resulting in EAE. Alternatively, T cell receptor transgenic T cells specific for a myelin peptide can be used. For example, CD4 T cells from a T cell receptor transgenic B10.PL mouse in which all the CD4 T cells recognize myelin basic protein (MBP) Ac1-11 peptide can be activated *in vitro* and injected into naïve B10.PL mice, resulting in classical EAE (33–35). In EAE, both myelin-specific Th1 and Th17 cells contribute to pathogenesis, and both cell types have been implicated in MS (35, 36). While myelin-specific T cells can be found in both healthy individuals and MS patients, myelin-reactive CD4 T cells from MS patients have an activated and/or memory phenotype, whereas those cells are naïve in healthy individuals (37–41), supporting the idea that myelin-specific CD4 T cells are contributing to disease pathology in MS.

## Physiology of Vitamin D

**Figure 1:**
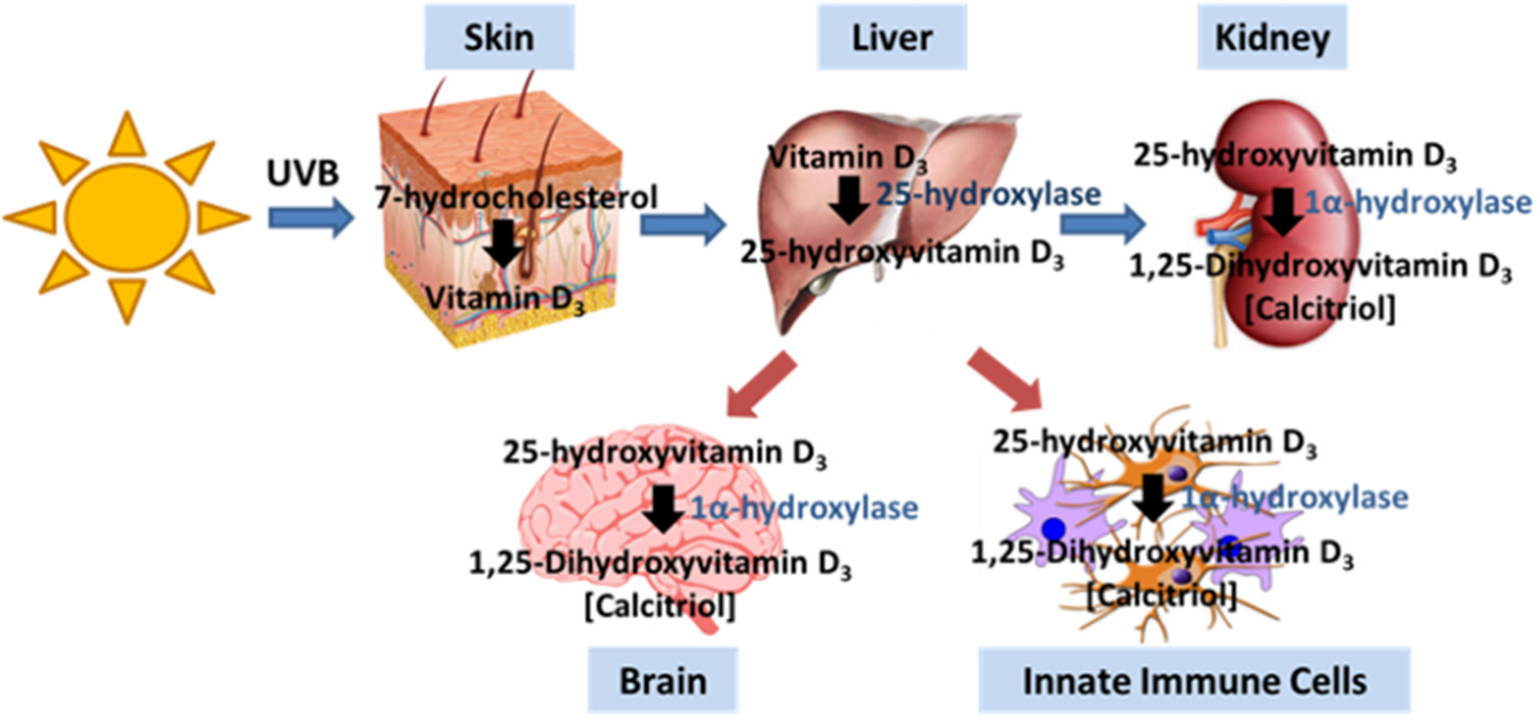
Vitamin D production pathway. Vitamin D3 is produced in the skin by UVB irradiation. Typically, the liver and kidney generate intermediates that ultimately generate calcitriol, the active form of VitD. The brain and immune cells also express the enzyme that allows for the generation of calcitriol.

For most people, exposure to sunlight is their major source of VitD. Ultraviolet B (UVB) photolyses 7-dehydrocholesterol to pre-vitamin D3 in the epidermis, which then isomerizes to vitamin D3 (Figure 1). VitD can be also obtained from diet through ingestion of vitamin D2 (ergocalciferol) derived from plants, colecalciferol supplements/fortified foods and oily fish. Vitamin D3 in the body then undergoes a series of hydroxylations, first to 25-hydroxyvitamin D3 (25(OH)D3) in the liver, the main circulating form of the vitD with relatively long half-life, and then to biologically active hormone 1,25-dihydroxyvitamin D3 (1,25(OH)2D3, also known as calcitriol) in the kidney (42). 1,25-dihydroxyvitamin D3 is the ligand for VitD receptor (VDR), a member of the nuclear receptor family of transcription factors which activates or represses the expression of many genes (43), and exerts rapid non-genomic effects via the membrane VDR (44).

**Figure 2:**
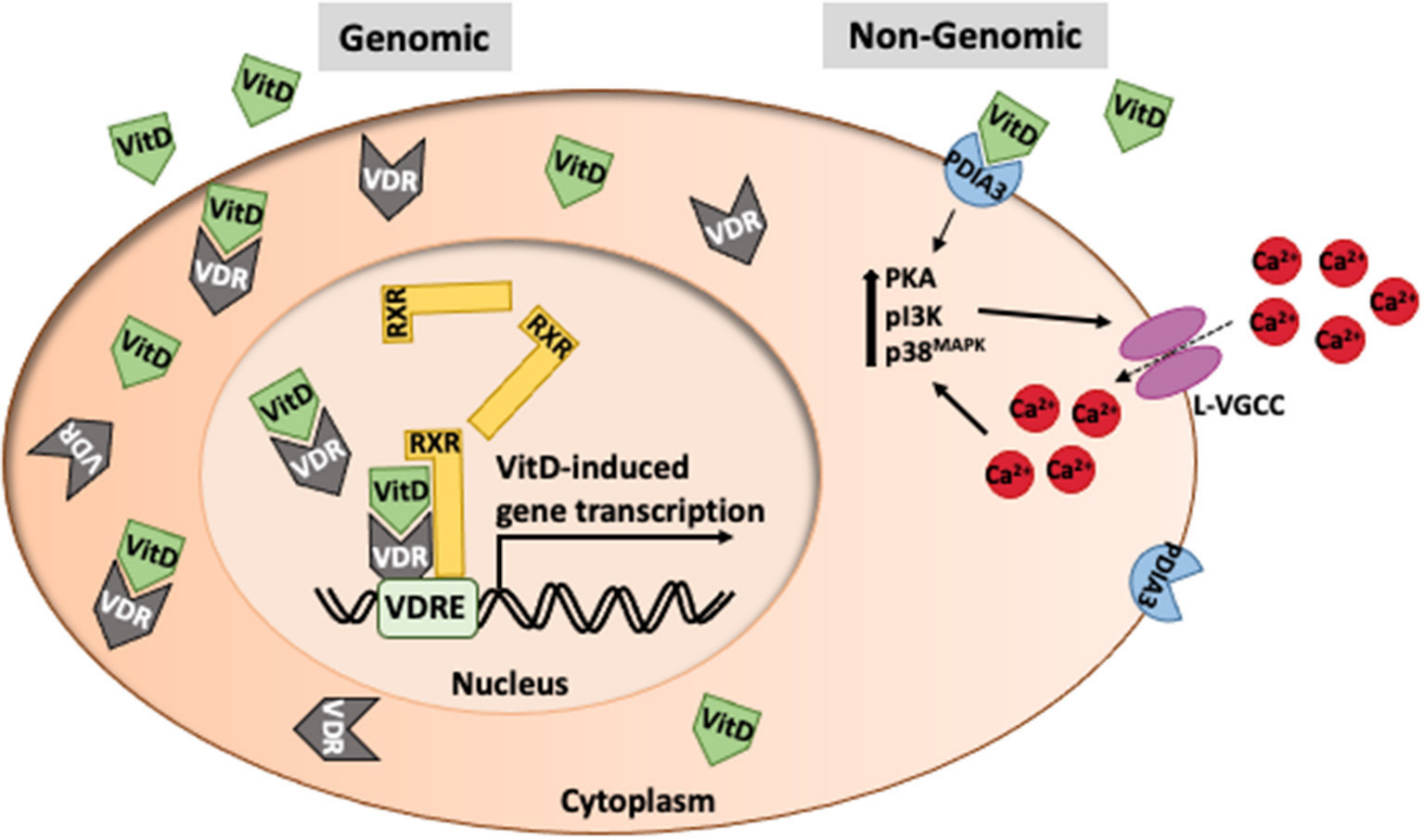
Intracellular function of vitamin D. VitD typically acts as a transcription factor in association with retinoid-X-receptors (RXR) to mediate gene transcription at VitD response elements in promoter regions of genes. However, VitD can have immediate effects on cell function (non-genomic) via interaction with PDIA3 that leads to changes in calcium transport.

VitD primarily acts as a hormone that regulates gene transcription. VitD enters cells using carrier proteins or diffusion where it can bind VDR in the cytoplasm (Figure 2). VitD/VDR complexes are translocated to the nucleus where VDR dimerizes with retinoid X receptors (RXR). VDR/RXR complexes bind to VitD response elements which are present in nearly 1,000 genes, thus playing a major transcriptional role in many cell types. There are non-genomic roles for VitD which occur within minutes, far too soon to be mediated by altered gene expression. The most noteworthy non-genomic effect of VitD is calcium regulation. VitD binds to protein disulfide isomerase family A, member 3 (PDIA3), resulting in the upregulation of PKA, pI3K and p38MAPK which contribute to the intracellular flux of calcium (Figure 2). While changes in intracellular calcium may be independent of VDR engagement, calcium homeostasis is affected by VDR signaling as seen in people with type II genetic rickets and VDR-deficient mice which have severe hypocalcemia (45–48).

Although the best-known function of VitD is to regulate calcium physiology, it also has important effects on the development and function of CNS. Neurons and microglia express VDR. In addition, they can directly metabolize 25(OH)D3 because they express 1-α hydroxylase (49). 1,25(OH)2D3 has been shown to regulate glial cell line-derived neurotrophic factor (GDNF) (50) and nerve growth factor (NGF) (51) expression. The ability of 1,25(OH)2D3 to regulate certain neurotrophic factors and influence inflammation has led to the hypothesis that 1,25(OH)2D3 is neuroprotective (52). In fact, it has shown a reduction in ROS induced cell death and increased anti-oxidant species in glia cell by 1,25(OH)2D3 (53). VitD insufficiency is associated with several other neurological disorders beside MS, including Parkinson disease, schizophrenia, depression and cognitive decline (54), suggesting its essential role in maintaining normal CNS function.

How much VitD is optimal is somewhat controversial. The most commonly published normal range for blood VitD levels is 20–40 ng/mL (50–100 nmol/L) with levels below 20 ng/mL (50 nmol/L) considered VitD deficient. The Endocrine Society considers VitD levels of 20–29 ng/mL (50–74 nmol/L) to indicate VitD insufficiency, and that VitD levels should be 30-100 ng/mL (75–250 nmol/L) for optimal health benefit (55). Based on these guidelines, it is estimated that 30–50% of Americans may be VitD deficient. A New England Journal of Medicine article suggested that VitD deficiency may be overstated, and perhaps our current metrics for VitD-deficiency are incorrect, because of misinterpretation of the Institute of Medicine's reference values (56). It should be noted that most of the studies evaluating VitD levels in health are based on intestinal uptake of calcium and bone health, so it remains unclear what the optimal dose may be for optimal overall health.

## Vitamin D and Ms

One of the strongest correlates of latitude is the duration and intensity of sunlight, and the synthesis of ViD is subsequently affected by ultraviolet B (UVB) radiation. The incidence gradient according to latitude and the effect of migration within genetically uniform groups can be explained by VitD— as the link between latitude and MS risk. The VitD hypothesis is supported by studies of sunlight exposure history. The seasonal fluctuations in VitD levels resulted in decreased VitD concentrations in *utero*, which may contribute the month-of birth effect in MS (57). While not all studies are in agreement, a large meta-analysis found that individuals born in the Spring have a significantly higher risks of developing MS compared to individuals born in the fall (58–60). Insufficient maternal 25-hydroxyvitamin D during early pregnancy is associated with a 2-fold increased risk of MS in offspring (61). Similarly, a Danish study used dried blood spot samples collected near the time of birth to measure VitD in individuals who later developed MS and matched controls (62). Neonatal VitD levels were inversely associated risk of developing MS, supporting the notion that maternal VitD levels may be important to prevent MS in children. Higher sun exposure during childhood (age of 6–15 years) was shown associated with reduced MS risks (63). Time spent on outdoor activities during childhood and adolescence (significant for age of 6–20 years) in the summer was inversely related to the risks, whereas there was no such effect in the winter (64). A study of monozygotic twins who were discordant with MS has shown that twins with MS reported significantly lower levels of childhood sun exposure than their healthy sibling (65). However, sunlight may have benefits to prevent CNS autoimmunity beyond VitD. A study in EAE found that UV light suppressed EAE independent of VitD and VDR (66).

Further evidence for the VitD hypothesis comes from the studies of dietary VitD intake. At high latitudes, prevalence of MS was lower than expected in populations with high consumption of VitD-rich oily fish (67). A 40% reduction in MS risks was found among women who used supplemental VitD, compared with women who did not use supplements (68). Lastly, a study directly measured the circulating 25(OH)D3 (the circulating form of VitD) concentrations in individuals who served in the US military, and concluded that serum level of 25(OH)D3 in healthy young white adults is an important predictor of their risk of developing MS (69). These epidemiology studies (latitude, migration, history of sunlight exposure, VitD intake and serum concentration of VitD) give credibility to the hypothesis that VitD, especially in early life, has protective effect against MS development. Nevertheless, due to often confounding variables in epidemiology studies, prospective experimental studies are needed to validate the effect of VitD in determining MS risks. A mendelian randomization study in which single nucleotide polymorphisms associated with 25-hydroxyvitamin D were identified and analyzed in the International Multiple Sclerosis Genetics Consortium found that there was a significant increased susceptibility to MS in individuals with a genetically lowered level of 25-hydroxyvitamin D (70). This genetic study supports the epidemiology data that optimal VitD levels are protective against the development of MS. Interestingly, a study of polymorphisms in the VitD-binding protein found an association with MS risk in whites, but not blacks or Hispanics, indicating that VitD may not be a significant risk factor in all ethnicities (71).

After disease onset, VitD also acts in modulating MS clinical course. Serum concentrations of 25-hydroxyvitamin D3 in MS patients were lower during relapses than during remissions (72), and correlated inversely with disease severity (73) and frequency of relapse (74, 75). Although these results might indicate lower sun exposure in patients with severe MS rather than a beneficial effect of VitD, convincing studies with EAE have demonstrated the immunomodulatory effect of VitD in inflammatory CNS disease. Expression of VDR has been described in immune cells, including dendritic cells, macrophages and activated T and B cells (76). VitD supplementation clearly suppressed EAE preventively (77, 78) and therapeutically (79). Moreover, the therapeutic effects of VitD required VDR function in T cells (80), and were through promoting IL-4, TGF-β (81) and IL-10 (82) production, and inhibiting TH1 cells differentiation (83, 84). With these results established from EAE, experimental basis supports the beneficial role of VitD in modulating disease progression. Yet, there are numerous studies that indicate that VitD supplementation in MS patients has little, if any benefit to reducing symptoms (85). The SOLAR trial found that VitD supplementation (14,000 IU/d) in MS patients on interferon beta-1a was beneficial in reducing new lesions, but no change in progression of disability or annualized relapse rate was observed (86). In the 2-year CHOLINE trial, MS patients on interferon beta-1a were given 100,000 IU of oral cholecalciferol or placebo biweekly. The end point (changed in annualized relapse rate) was not met, yet there were positive benefits observed on imaging and the average EDSS score was significantly lower in the treatment group (87). Analysis of 12 random controlled trials evaluating VitD supplementation concluded that VitD supplementation had no significant benefit on relapse rates, progression of disability or MRI lesions (88). Thus, the benefit of VitD supplementation in MS patients is unclear, but given that most MS patients are VitD deficient, supplementation is prudent and likely benefits that overall health of the patients.

## Vitamin D and the Immune System

The first evidence that VitD may affect the immune system came from a study in 1983 in which VitD promoted the fusion of macrophages (89). In 1986, it was shown that VitD inhibited IL-2 production and proliferation by T cells (90), providing solid evidence that VitD had the capacity to regulate T cells. There is now substantial evidence that VitD is a major regulator of both innate and adaptive immune cells and influences the outcomes of infections, cancer and autoimmunity.

### Innate Immunity

**Figure 3:**
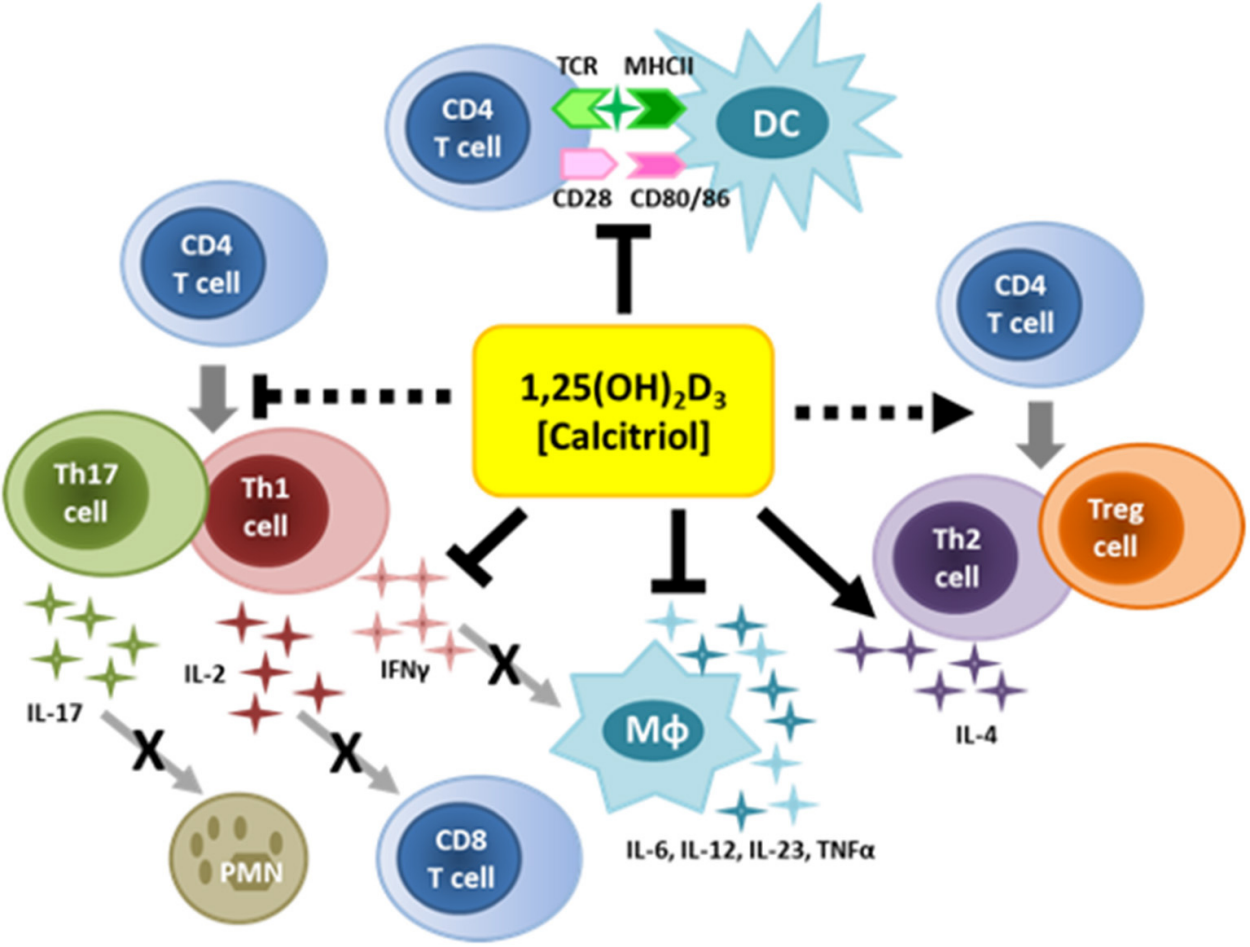
Immunoregulatory functions of vitamin D. VitD can regulate the function of numerous immune cells. VitD suppresses inflammatory cytokines and antigen-presentation by innate immune cells. VitD also suppresses T cell activation, and favors the generation of Th2 cells and Tregs.

Activated macrophages and monocytes upregulate expression of CYP27B1, the gene that encodes 1α-hydroxylase, the enzyme that converts 25-hydroxyvitamin D_3_ to 1,25-dihydroxyvitamin D_3_ (calcitriol), the active form of VitD (Figure 1), indicating that macrophages/monocytes have the capacity to increase VitD at site of inflammation (91). The local expression of VitD by activated macrophages/monocytes sets up an autocrine pathway since macrophages/monocytes express VDR, resulting in the production of anti-microbial products such as cathelicidin and defensins. Cathelicidin is particularly important against infections by destablilizing microbial membranes and disrupting viral envelopes (92–94). Equally important, VitD regulates the maturation and activation of macrophages and dendritic cells, compromising their ability to be effective antigen presenting cells (Figure 3). Upon TLR engagement, macrophages and dendritic cells upregulate MHCII, CD80/CD8, CD40 and cytokines which are critical to antigen presentation to T cells. VitD suppresses these molecules, promoting macrophages and dendritic cells that are immature and somewhat tolerogenic (95, 96). This suppression of macrophages and dendritic cells may be due to the suppression of toll-like receptors (97–99) or inhibition of IL-12 via NF-κB (100) mediated by VitD.

VitD affects the function of microglia which are known to secrete inflammatory mediators that contribute to myelin damage during CNS autoimmunity. Mice with EAE treated with calcitriol immediately following EAE induction have reduced microglia activation and oxidative stress, and less blood brain barrier permeability (101). Partial deletion of VDR in young mice attenuated microglia activation and reduced the incidence and severity of EAE (102). In various models of CNS diseases and injury, VitD has been shown to regulate microglia phenotype and oxidative stress (103–106). Thus, vitD appears to effectively skew microglia from a pro-inflammatory M1 phenotype to a reparative M2 phenotype, reducing inflammation and limiting demyelination.

### Adaptive Immunity

Both T cells and B cells can express VDR and respond to VitD (Figure 3). There is actually very little, if any, VDR expression on resting human T and B cells; however, VDR is rapidly upregulated upon activation (107–109). Similar to innate immune cells, activated T cells express CYP27B1 and can make active VitD. VitD suppresses T cell proliferation via reduced IL-2. In addition, VitD alters the differentiation of CD4 T cells by skewing CD4 T cells toward Th2 and away from Th1 and Th17, the phenotypes associated with MS (110, 111). Also of particular relevance to MS, VitD promotes the differentiation of Tregs (112), which are known to be defective in MS patients (113–118). The mechanism by which VitD promotes Treg development appears to be via altered APCs, since VitD added to human dendritic cells alters glucose metabolism favoring the differentiation of Tregs over Teff cells (119). VitD status in MS patients positively correlates with Treg function, supporting the observation the VitD promotes Treg development (120). VitD in association with CD46 was shown to promote Type I regulatory T cells (Tr1) cells in MS patients (121), indicating that optimal VitD may be an important component of immune regulation.

B cell differentiation and maturation into plasma cells is also regulated by VDR, thus affecting antibody production. In addition, VitD downregulates co-stimulatory molecules on B cells, similar to what is observed for macrophages and dendritic cells (122). Thus, optimal VitD may compromise the ability of B cells to act as antigen presenting cells. B cell –depletion therapies have been very beneficial in the treatment of MS. Given that the beneficial affects appear to be independent of antibody production, there is speculation and evidence that B cells are critical antigen-presenting cells in MS (123–125). An immune profile study on MS patients on B cell depletion therapy indicated that the T cell profile showed a favorable change, reflected by a reduction in memory T cells and an increase in Tregs (124, 125), consistent with the role of B cells as antigen-presenting cells. Thus, low VitD may enhance the ability of B cells to drive the activation and differentiation of T cells, increasing the probability developing MS.

## Vitamin D and the Central Nervous System

Substantial evidence indicates that VitD acts as a neurosteroid. VDR is expressed throughout the developing and mature brain, including the hippocampus, amygdala, hypothalamus, cortex and cerebellum (49, 126, 127), implicating VitD as an important modulator of gene expression throughout the CNS. Furthermore, 1α-hydroxylase and 25-hydroxylase are both expressed in the brain providing the critical enzymes to generate VitD locally (49, 128). While a major role of VitD is gene regulation, it also has non-genomic functions, particularly regulation of calcium signaling which is critical in normal cellular function.

### Vitamin D and Neurogenesis

VitD promotes cell differentiation and apoptosis which are critical for embryonic development. When VitD is removed during gestation in model systems, multiple regions of the brain have increased cell proliferation and decreased apoptosis, as well as enhance cell proliferation, leading to CNS anomalies (129–131). The increased proliferation was mediated by increased expression of cyclin genes which were regulated by VDR signaling (132), while the reduction in apoptosis may have been due to increased levels of BAX and Bcl-2 (131). Low VitD also leads to more neural stem cells which may be due to a loss of regulation of cell proliferation or a failure in neural stems to efficiently differentiate into neural cell progenitors (133). Changes in brain morphology have been observed in rodents with VitD deficiency (129, 134). In humans, VitD deficiency is associated with decreased brain volume and enlarged ventricles in older adults (135). *Ex vivo* studies illustrated that VitD inhibits the proliferation of hippocampal neurons, while promoting neurite outgrowth (130). Analysis of dopaminergic neurons found that VitD-deficiency reduced Nurr1 and P57kip2 gene expression during embryogenesis which are critical to the development and homeostasis of dopaminergic neurons (136). In addition, VitD has been found to regulate the expression of genes essential in the normal function of dopaminergic neurons (137, 138). Interestingly, while it appears that VitD promotes the differentiation of neurons, astrocyte differentiation appears to be impaired by VitD when using adult neural stem cells (139). VitD also promotes the differentiation of neural stem cells into oligodendrocytes (139), the myelinating cells of the CNS. Oligodendrocyte precursor cells fail to differentiate into oligodendrocytes when VDR signaling is blocked (140). These studies implicate VitD as an essential regulator of neuron and oligodendrocyte development. The signaling between neurons and oligodendrocytes is essential to the development of a properly myelinated CNS during early life, as well as remyelination that occurs following CNS damage.

### Functional Consequences of Low VitD in the CNS

In addition to the development and differentiation of CNS cells, VitD plays a role in their ability to function properly. The release of several neurotransmitters is affected by VitD. In dopaminergic neurons, VitD promotes the release of dopamine (141). VitD appears to regulate neurotransmitter synthesis, for example, VitD regulates the inhibitory neurotransmitter GABA, via upregulation of GAD65 and GAD67 (142–144). Neurotrophic factors, which are essential for CNS homeostasis and communication between cells in the CNS, are also regulated by VitD. VitD appears to induce nerve growth factor (NGF) expression in neurons (129, 135). Neural stem cells upregulate brain-derived growth factor (BDNF), Glia cell line-derived nerve factor (GDNF), and ciliary neurotropic factor (CNTF) in the presence of VitD (139). In astrocytes, VitD appears to regulate the expression of neurotrophin receptors, as well GDNF, NT-3 and NT-4 (145–147).

VitD also plays a role in neuronal plasticity. In cultured cortical neurons, VitD increased the expression of microtubule associated protein-2, growth-associated protein-43, and synapsin 1 which are important in synaptic vesicle transport and axonal growth (148). Low VitD during embryogenesis resulted in altered expression of proteins important in cytoskeletal integrity, organelle transport, and synaptic plasticity (149, 150), suggesting that VitD is critical to the normal development and function of the CNS.

### Calcium Regulation

VitD plays a vital role in regulating calcium levels in the CNS which is particularly important given that high levels of calcium are neurotoxic. In neurons, VitD modulates L-type voltage-gated calcium channels by downregulation of A1C subunits (151). Mice lacking VitD have upregulated L-type voltage-gated calcium channels and elevated calcium influx in neurons (152) (Figure 2). *In vitro* treatment of neurons with VitD downregulated L-type voltage-gated calcium channels and protected neurons from excitotoxicity (153). VitD was shown to very rapidly increase the uptake of extracellular calcium via L-type voltage gated calcium channels (154). Since this occurred within a few minutes, it was clear that the effect was independent of gene transcription. The increase in calcium influx was dependent on the PKA, pI3K, and p38^MAPK^. The non-genomic effects of VitD have largely been attributed to VitD interaction with PDIA3 (also known as 1,25D_3_-Marrs) on the plasma membrane (155, 156). In addition, VitD regulates the expression of numerous genes associated with calcium homeostasis vital to the proper regulation of calcium in the CNS and other tissues (150, 154, 157).

### Vitamin D and Neuroprotection

Epidemiological data indicate that VitD has neuroprotective properties. Optimal VitD levels during early life appear to be important to minimize the risk of several psychiatric and neurodegenerative diseases. Schizophrenia, depression and autism spectrum disorders have all been associated with low VitD, particularly during embryogenesis and infancy (158–162). There is an inverse correlation between Parkinson's disease risk and VitD levels (163, 164). Given that VitD protects dopaminergic neurons by upregulating genes numerous genes associated with the function of dopaminergic neurons (137, 138), it is logical that VitD may be a critical factor in minimizing the risk of developing Parkinson's disease. Similarly, Alzheimer's disease patients tend to have low serum VitD levels compared to matched healthy controls (165). The risk of dementia and symptoms of neurodegenerative diseases, such as cognitive and memory impairments and impaired motor function, increases with low serum VitD levels (166–169). Serum VitD deficiency is linked to greater infarct volumes, increased overall stroke severity, and worse long-term outcomes in stroke patients (170–172). Impacts on the risks and outcomes in these neurological conditions are clearly multifactorial, but it stands to reason that VitD plays a role in susceptibility and outcome.

The neuroprotective properties of VitD take effect though several mechanisms. Direct neuroprotective action of VitD is associated with the regulation of neurotrophic factors and reduction in oxidative stress. Neurotrophic factors are critical for the differentiation, survival and maintenance of neural and glial cells. VitD stimulates expression of nerve growth factor (NGF), brain-derived neurotrophic factor (BDNF), glial cell line-derived neurotrophic factor (GDNF) and neurotrophin-3 (NT3) (173). Neurotrophic factors downregulate their expression into adulthood, therefore remaining levels have critical cell maintenance functions. Reduction in VitD-assisted neurotrophic factor expression due to deficiency may leave neurons more vulnerable to insult.

Neurons are particularly susceptible to oxidative damage because of increased oxygen consumption, bi-products of neurotransmitter production, excitotoxicity and high overall lipid content (174). Additional neuroinflammation will increase the reactive oxygen species (ROS) load. Adequate VitD levels downregulate intracellular oxidative-stress related activities, while suboptimal levels result in increased oxidative damage and neuronal apoptosis (175). Increased reactive oxygen production has been implicated in the pathogenesis of multiple neurodegenerative conditions, including Parkinson's disease (176), Alzheimer's disease (177), Huntington's disease (178), stroke (179) and Multiple Sclerosis (180), and suboptimal serum VitD levels have been linked to these conditions. VitD is a potent regulator of the nuclear factor erythroid-2-related factor 2 (Nrf2) antioxidant pathway in neurons and glial cells, and intracellular Nrf2 levels are inversely correlated with the accumulation of mitochondrial ROS. Within the CNS, upregulation of Nrf2 target genes superoxide dismutase (SOD), catalase (CAT) and heme oxygenase-1 (HMOX1) to make neurons more resistant to oxidative insults (181). Furthermore, neurotrophic signaling pathways, such as the BDNF-TrkB pathway that is essential for mature neuron survival and normal function, also signal Nrf2 activation (182, 183). VitD then has double the influence on neuronal survival – first in neurotrophic action by increasing levels of BDNF, and second in oxidative defense by direct activation of Nrf2. The neuroprotective properties of VitD center around its antioxidant function, and in conjunction with neurotrophic factor expression, likely enhances neural defense and repair mechanisms.

### Vitamin D as a Neuroprotective Agent in MS Through Antioxidant Pathways

Oxidative stress and mitochondrial dysfunction are prominent features of MS. T cells can produce ROS and T cell activity and proliferation are influenced by ROS, adding an increased level of complexity to the impact of ROS in MS (184). Activated microglia and macrophages are the major contributors to the elevated ROS observed in the disease. These cells produce oxidating radicals such as superoxide, hydrogen peroxide and nitric oxide with the help of ROS-generating enzymes, such as myeloperoxidase, NADPH oxidase and nitric oxide synthase. ROS have shown to mediate demyelination in both MS and its animal models (184, 185). Studies in postmortem brains of MS patients have identified myeloperoxidase expression in activated macrophages and microglia near lesions. Elevated expression of myeloperoxidase was detected in demyelinated regions of postmortem MS brain homogenates when compared to unaffected regions from the same individual (186, 187). Other markers of oxidative damage, such as 4-hydroxy-2-noneal (4-HNE), produced by lipid peroxidation of cell membranes, and nitrotyrosine, the product of nitric oxide and superoxide reactions, increase and accumulate in macrophages and astrocytes in MS lesions (188–190).

ROS in later stages of MS stems from mitochondrial dysfunction within neurons themselves. Mitochondrial dysfunction and associated ROS have been implicated in non-inflammatory mediated axonal degeneration that occurs with chronic demyelination. It is believed that mitochondria become taxed after sodium channel redistribution in response to demyelination. Sodium channel redistribution causes large influxes of sodium, taxing the ATP dependent sodium-potassium pump (191). Increased ATP needs trigger mitochondria production and proliferation, resulting in increased ROS (192, 193). Notably, increased mitochondrial heat shock protein 70, a marker of mitochondrial stress, has been observed in astrocytes and axons within MS lesions (192). There is some controversy regarding whether increased ROS from mitochondria exists primarily due to mitochondrial proliferation and ATP production after demyelination (chronic injury) or if mitochondria actually acquire oxidative damage during the inflammatory stage of the disease (acute injury) (194).

Antioxidant enzymes are the endogenous ROS defense system in the CNS. ROS exposure activates Nrf2, which then translocates to the nucleus and activates antioxidant response element promoters (ARE) for antioxidant enzyme production. Expression of hundreds Nrf2 responsive antioxidant genes have already been identified (195). Numerous studies have suggested a role for Nrf2 inactivity in the pathogenesis of MS. Nrf2 knock-out EAE mice experience more rapid disease onset, a more severe clinical course, increased glial activation, increased pro-inflammatory cytokine expression and increased axonal degeneration compared to Nrf2 inclusive controls (196–198). Conversely, increasing the activity of Nrf2 in the CNS of EAE mice lessened the clinical severity (199). In postmortem brain tissue of MS patients, NRF2 is strongly upregulated in active MS lesions and expression is most pronounced in degenerating neurons and glial cells, including oligodendrocytes (200, 201).

NRF2 activity is already relevant in MS clinical treatment. Both VitD and dimethyl fumerate (DMF or Tecfidera™) are NRF2 activators. DMF treatment is an approved oral RRMS therapy known to reduce disease activity and progression, and accomplishes these outcomes via immunomodulatory and neuroprotective mechanisms (202–207). VitD and DMF both signal through the NRF2/KEAP1 pathway to generate antioxidant action and specifically increase glutathione signaling for neuroprotection. DMF and VitD can also exert protective effects by reducing proinflammatory cytokine expression and increasing neurotrophic factor expression (208). DMF and VitD derivatives have demonstrated a cooperative effect on increased VDR expression and Nrf2 activity that limit leukemia progression (209). Although mechanisms of action overlap, DMF is a safer therapeutic option for avoiding calcemic toxicity that can occur with long-term use of high levels of VitD. In fact, excess VitD can exacerbate EAE, emphasizing the point that a measured approach to VitD-based therapies is critical (210). It should also be noted that melatonin which is produced during the dark has similar anti-oxidant properties as VitD in EAE (211, 212). There is contradicting data on the relationship between melatonin and VitD, yet both appear to be beneficial in reducing CNS inflammation. Thus, the interplay between appropriate sunlight to optimize VitD levels and appropriate darkness to optimize melatonin to maintain circadium rhythm should be considered in strategies to prevent and/or treat MS. It is unlikely that DMF mimics every function of VitD, but similarities in function between DMF and VitD suggest that VitD has a critical role as an endogenous neuroprotective mediator in MS.

An important consideration is the temporal influence of VitD, NRF2 activity, and the endogenous antioxidant system during the course of MS. Upregulated expression of NRF2 in MS brain lesions suggests that the NRF2 pathway is already highly active in distressed cells (200, 201) and it appears that endogenous antioxidant mechanisms are not enough to halt demyelination and axonal degeneration at end stages of disease. However early in the disease, VitD and NRF2 signaling may have increased potential for neuroprotection because less neuroinflammatory-induced oxidative damage has occurred. Understanding the neuroprotective potential of VitD in early stages of MS is complicated by the striking frequency of VitD deficiency in patients at the time of diagnosis. Therefore, whether preventing VitD deficiency prior to disease onset can enhance neuroprotection and alter disease progression warrants further investigation.

## Immunoregulation Vs. Neuroprotection

MS is a complex disease in which immune and nervous system components interact to form and sometimes, resolve CNS inflammatory, demyelinating lesions. Genetic studies have largely implicated immune genes as susceptibility factors, supporting the hypothesis that MS is an immune-mediated disease and that the CNS is the unfortunate target of the aberrant immune response. There is significant data to support that VitD has a profound immunoregulatory role on both innate and adaptive immune cells, indicating that low VitD may alter normal immune regulation leading to autoimmunity. Given that the CNS is the sole target of the aberrant immune response in MS, it is important to consider if the CNS is somehow more vulnerable to inflammation in some individuals that may make them at an increased risk of developing MS. While the answer is still unknown, the literature clearly indicates that VitD impacts CNS development and function.

The epidemiology studies indicate that optimal VitD is particularly important during embryogenesis and childhood in determining risk of developing MS. During childhood, our immune system is repeatedly being challenged by pathogens, most of which are cleared with minimal clinical consequences. Some childhood viruses, such as Epstein-Barr, varicella zoster, and some strains of influenza, can infect neurons, yet they typically do not cause clinical CNS manifestations. Even in the absence of CNS clinical signs of illness, do these viruses affect the CNS differently in children with low VitD? Our recent study in which partial VDR deletion was induced in young mice specifically in neurons resulted in an enhanced susceptibility to EAE in adult mice, suggesting that low VitD signaling in neurons in early life may increase the vulnerability of the CNS to inflammation (102). There is substantial evidence that viral infections are affected by VitD levels. Of particular interest in MS is EBV which has been long speculated to be a vital risk factor for the onset of disease and this has been confirmed in a new study of >10 million young adults (213). While many theories have been explored, a recent study identified EBV infection as a precipitating factor that may be driving molecular mimicry (214–217). EBV antibody titers are negatively correlated with VitD levels in MS patients (218) suggesting that these two environmental factors may be synergistic. EBV is also associated with activating endogenous retroviruses (ERVs) and ERV levels in MS plaques correlates with disease activity (219). These ERVs may act as novel antigens that drive CNS inflammation. VitD supplementation mitigates EBV reactivation (220), which may in turn limit ERV activation and the associated inflammation. In a humanized mouse model, it was shown that HLA-DR15-restricted T cells fail to control EBV infection, suggesting that there is potential relationship between the strongest genetic factor (HLA-DR15) and EBV with respect to MS risk (221). The relationship between ERVs, VitD and MS has become of increasing interest and some speculate that EBV may be the missing link between ERVs and VitD that trigger the development of MS (222). While many autoimmune diseases have been associated with low VitD to some extent, the epidemiology data in MS is far more convincing suggesting that low VitD is likely increasing the risk of developing MS due to the negative impact on immune regulation and CNS homeostasis.

A recent study of >1,900 subjects demonstrated that sun exposure negatively correlated with development of MS, and high VitD levels (>30.31 ng/mL) in MS patients reduced the risk of relapses and accumulation of disability (223). The evidence that VitD level is important in risk of developing MS and disease severity appears well established, yet questions still remain as to whether VitD supplementation is beneficial to patients with MS. Since most MS patients have low VitD levels, supplementation is now common practice. Perhaps the more important question is how do we prevent low VitD? Although VitD is currently a common food supplement in many countries, it is unclear whether we are doing enough to ensure that children are getting sufficient VitD. Rickets is rare in countries in which food is supplemented with VitD, indicating that the levels of VitD provided via food supplementation is sufficient for bone health. However, it is unclear whether these levels of VitD are adequate for optimal neuroprotection, given that countries like the United States still have a high incidence of MS. Additional VitD supplementation may be essential for children in higher latitudes where sunlight is very limited for several months each year, and perhaps in children with a family history of MS in which genetic risks are highest. We should also balance pros and cons of sunscreen which reduces UVB induced VitD synthesis by 95% and may negatively impact health of our immune and nervous systems. Sunlight remains the best source of VitD so ensuring that children play outside daily may be the best solution to the epidemic of low VitD.

## Author Contributions

AL-R, SG, PL, and ES each reviewed the literature and wrote sections of the manuscripts. All authors contributed to the article and approved the submitted version.

## Conflict of Interest

The authors declare that the research was conducted in the absence of any commercial or financial relationships that could be construed as a potential conflict of interest.

## Publisher's Note

All claims expressed in this article are solely those of the authors and do not necessarily represent those of their affiliated organizations, or those of the publisher, the editors and the reviewers. Any product that may be evaluated in this article, or claim that may be made by its manufacturer, is not guaranteed or endorsed by the publisher.
